# Game-matching background music has an add-on effect for reducing emotionality of traumatic memories during reconsolidation intervention

**DOI:** 10.3389/fpsyt.2023.1090290

**Published:** 2023-02-15

**Authors:** Che Jiang, Wei Chen, Ling Tao, Jiajia Wang, Kuihong Cheng, Yibo Zhang, Zijuan Qi, Xifu Zheng

**Affiliations:** ^1^School of Psychology, South China Normal University, Guangzhou, China; ^2^Department of Neurosurgery, General Hospital of Southern Theatre Command, Guangzhou, China; ^3^Computer Engineering Technical College, Guangdong Polytechnic of Science and Technology, Guangzhou, China; ^4^Department of Neurology, General Hospital of Southern Theatre Command, Guangzhou, China

**Keywords:** cardiopulmonary resuscitation, intrusive memories, visuospatial game, reconsolidation intervention, flow experience

## Abstract

**Introduction:**

Hospital is a stressful place of employment, and a high proportion of healthcare workers, especially the ICU (Intensive Care Unit) nurses were found to be at risk of PTSD. Previous studies showed that taxing working memory through visuospatial tasks during the reconsolidation process of aversive memories can reduce the number of intrusions afterwards. However, the finds could not be replicated by some researches, indicating there may be some boundary conditions that are subtle and complex.

**Methods:**

We performed a randomized controlled trial (ChiCTR2200055921; URL: www.chictr.org.cn). In our study, a series of ICU nurses or probationers who performed a cardiopulmonary resuscitation (CPR) were enrolled and instructed to play a visuospatial music tapping game (“Ceaseless Music Note”, CMN; Beijing Muyuan Technology Co., Ltd., Beijing, China) at the fourth day after CPR. The numbers of intrusions each day were recorded from the first to the seventh days (24 h×6 day), and the vividness and emotionality of CPR memories were rated at the 4th and 7th days. These parameters were compared between different groups (game with background sound; game with sound off; sound only; none).

**Results:**

The game-matching background music can have an add-on effect for single tapping game with no sound in reducing the emotionality of previous aversive memories.

**Discussion:**

We proposed that flow experience (the subjective experience of effortless attention, reduced self-awareness, and enjoyment, and may be induced by optimal skill-demands compatibility in challenging tasks) as a key boundary condition for successful reconsolidation intervention.

**Clinical trial registration:**

www.chictr.org.cn, identifier: ChiCTR2200055921.

## 1. Introduction

Exposure therapy represents a major cognitive behavioral treatment for anxiety disorders like posttraumatic stress disorder (PTSD) (1, 2). Through repeated exposing of individuals to the feared objects or situations in a safe environment, the fear is to be reduced. Yet, despite its efficacy, exposure therapy has some shortcomings including high drop-out rate [estimated to be 36% among individuals with PTSD (3)] and relapse rate [the estimated return of fear ranges from 19% to 62% (4)], and it is not always effective (5, 6). Specifically, the cause of high relapse rate is considered owing to the mechanism underlying the exposure therapy, which relies on the competition between the memory of the past distressing experience and the newly-formed memory of safety. Accordingly, efforts have been made to directly change the original distressful memory to reduce the possibility of relapse. Over the past two decades, abundant studies have demonstrated that under certain retrieval conditions, even long-established and consolidated memories can be induced to a transient labile state, during which they are susceptible to interference before being reconsolidated (7–9). Thus, the process of reconsolidation intervention comprised two dissociable stages: a prior stage of retrieval to induce destabilization of the targeted memory and a following stage of restabilization in an updated form (10). Behavioral therapies and pharmacological agents are two ways of interference in the second stage.

Behavioral ways include exposure therapy (11, 12) and taxing working memory through visuospatial tasks [e.g., concealed complex pattern tapping (13), eye movement desensitization and reprocessing, and the computer game Tetris (14)]. It is important to note that these behavioral procedures targeted different aspects of the memory. In the Tetris game, different shaped blocks fall from the top to the bottom of the screen one at a time, and players gain scores by using arrow keys to rotate and move each block when it is falling to form a full horizontal line. All full lines of blocks are removed. The speed of block falling increases gradually, and the game ends when the blocks are stacked to the top of the screen. Playing the Tetris game was recently found to be a simple and easily-accessible procedure that can effectively reduce the number of intrusive memories, which is the “core clinical feature” [a term forwarded by Kupfer and Regier (15)] of PTSD and acute stress disorder (16), of both experimental traumas (17–19) and real-world traumas (20–22). The theoretical basis for the effectiveness of visuospatial tasks is that most intrusive memories are visual imageries (23, 24), thus can be selectively disrupted by taxing working memory *via* competition for limited cognitive resources (25–27) when that memory is labile. However, this theory was challenged by some researches with negative results (28, 29), and also the effects of Tetris game play could not be replicated by some researchers (30, 31). These results indicate that there may be some boundary conditions that are subtle and complex. One of the proposed factors that may be critical for the psychobiological impact of gameplay is “flow experience” (29), which refers to the subjective experience of effortless attention, reduced self-awareness, and enjoyment, and may be induced by optimal skill-demands compatibility in challenging tasks (32).

In the studies of auditory intrusions, results showed that taxing working memory during memory recall would result in decreases in vividness and emotionality, and this effect may be modality matching (33, 34) as well as cross modal (i.e., a mismatch between visual modality of the intervention and the auditory modality of the recalled memory) (35–37). Thus, one would presume that adding auditory intervention may increase the interventional effect of visuospatial tasks.

Hospital is a stressful place of employment, and a high proportion of healthcare workers were found to be at risk for PTSD (38). Specifically, the ICU nurses are frequently exposed to work-related stresses, such as end-of-life issues, performing cardiopulmonary resuscitation (CPR), postmortem care, and prolonging life by artificial support to critically ill patients (39). They are predisposed to develop work-related psychological disorders including PTSD, the prevalence of which is approaching 25% (39, 40). These findings underlined the importance of developing preventive therapies and treatment of PTSD for ICU nurses. This will not only improve their mental health, but also improve their job satisfaction, and reduce the growing exodus of nursing from their profession (40). It is estimated that between 40 and 84% of all CPR attempts in ICU result in immediate or imminent death of the patient within 24 h (41–45). During CPR, the physical signs and sounds (e.g., color changes, gasping, emesis, and indignities suffered by patients) are perceived as stressful by nurses (46). Unsuccessful CPR represents one of the major traumatic events for ICU nurses, and may eventually lead to PTSD (39, 47, 48).

## 2. Theory

In the present study, we conducted a randomized controlled trial with a series of ICU nurses or probationers who performed a CPR in <12 h. We tested the hypothesis that: (a) a new visuospatial music tapping game (“Ceaseless Music Note”, CMN; Beijing Muyuan Technology Co., Ltd., Beijing, China) applied at 3 days after CPR can reduce the number of intrusive memories, as well as the vividness and emotionality of CPR recalls; (b) background music can have an add-on effect in this reconsolidation intervention, especially for auditory intrusions, either by exerting additional cognitive taxing on players or by the state of flow experience.

## 3. Materials and methods

**Figure 1:**
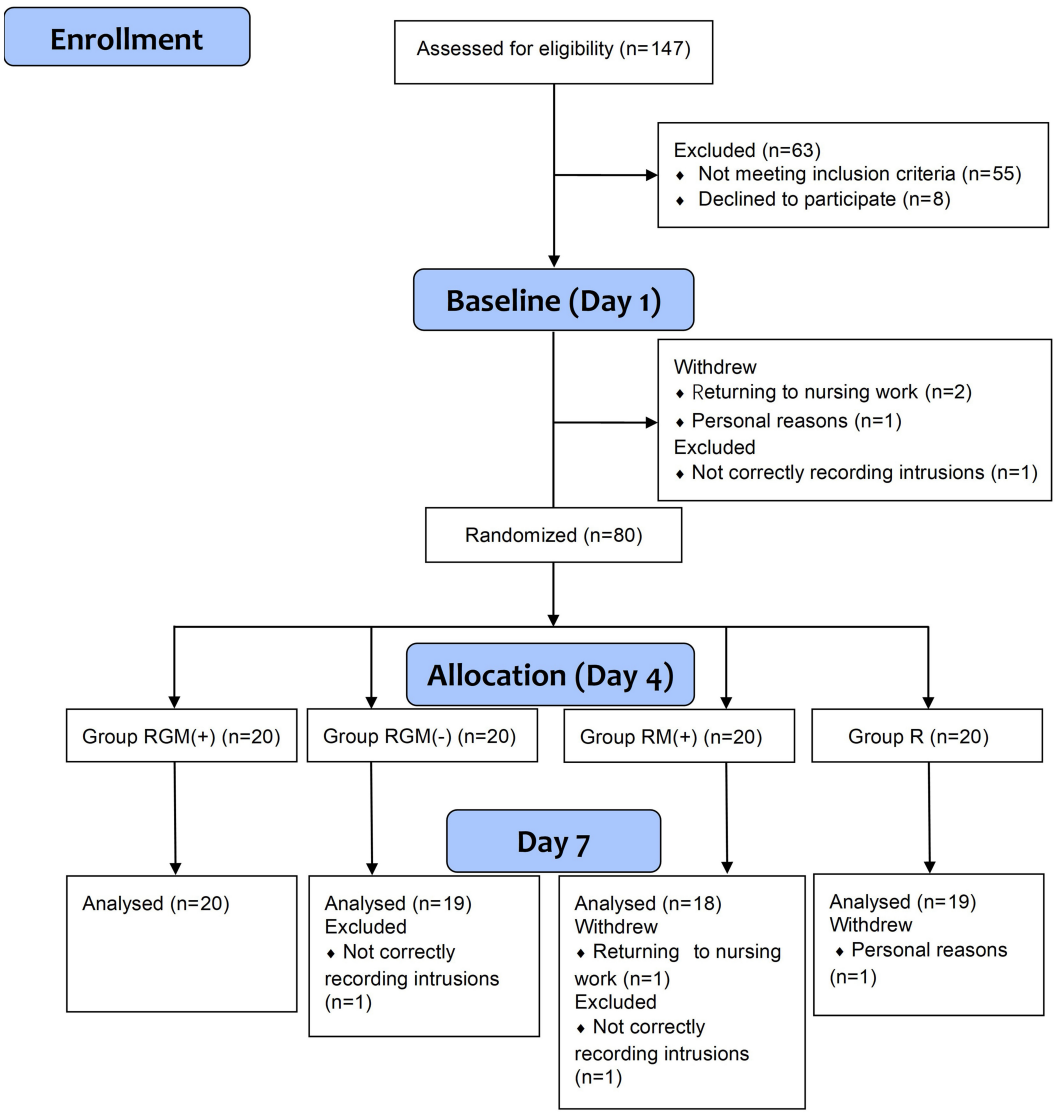
Flow diagram of this study.

This was an exploratory open-label randomized controlled trial (ChiCTR2200055921). The investigation was carried out in accordance with the latest version of the Declaration of Helsinki, and was approved by The Ethics Committee of General Hospital of Southern Theatre Command. This study's design and hypotheses were preregistered; see [www.chictr.org.cn]. The data was shared on the website www.medresman.org.cn. The flow diagram was shown in Figure 1.

### 3.1. Participants

Female nurses or probationers who had participated a CPR during the previous 12 h in the General Hospital of Southern Theatre Command were screened. Inclusion criteria were as follows: nurses participating CPRs in our hospital within 12 h; after CPR, they can change from clinical nursing work to clerical work for the next 6 days; female sex; aged between 18 and 40 years; no acute serious illness; mother tongue is Chinese; signed written informed consent. Exclusion criteria were as follows: frequent CMN players; have experienced other CPRs in the past 1 month; participate in CPR twice or more during the trial period; failure to sleep on time due to CPR; history of mental illness; alcohol or drug abuse; have experienced severe traumatic events recently; hearing impairment. Participants who did not complete the whole process of this trial were further excluded. Potential participants were identified by a staff of our research team. Eligibility was assessed by face-to-face interview. All participants were asked to keep to a regular daily schedule during the trial period.

Sample size was calculated based on results of previous studies (49). We set α = 0.05, β = 0.1, Ψ = 2.17, ${\bar{\text{X}}}_{1}$ = 10, ${\bar{\text{X}}}_{2}$ = 8, ${\bar{\text{X}}}_{3}$ = 5, ${\bar{\text{X}}}_{4}$ = 5, S_1_ = 6, S_2_ = 5, S_3_ = 5, S_4_ = 4. By referring to the sample size estimation table, we got a total sample size of n=80 for four parallel groups.

Eighty female nurses were randomized. One participant withdrew from the trial due to accidental reasons that they had to return to nursing work. One participant withdrew from the trial for personal reasons. Two participant was excluded for not correctly recording intrusions. Thus, a total of 76 participants were included for the final analysis.

### 3.2. Randomization and covert grouping

The participants were randomly assigned in a 1:1:1:1 ratio to four groups by random number table: reminder plus playing CMN with background music [group RGM (+)], reminder plus playing CMN with background music off [group RGM (-)], reminder plus only background music [group RM (+)], and only reminder (group R).

### 3.3. Task and measures

#### 3.3.1. Baseline characteristics

Age, the second edition of Beck Depression Inventory (BDI-II) (50), State-Trait Anxiety Inventory (STAI-T) (51), and negative emotion caused by CPR were recorded.

The Chinese version of BDI-II was used to measure depressive symptoms. It contains 21 items with each item evaluated ranging from 0 to 3. Higher total scores indicate higher severity of depression. Total scores of ≤ 10 indicate no depressive symptoms. The BDI-II has high internal consistency in clinical outpatients (α = 0.92) and student samples (α = 0.93) (50).

The Chinese version of the trait subscale of STAI (STAI-T) was used to evaluate trait anxiety. It consists of 20 items, and each item is rated from 1 (not at all) to 4 (extremely). Higher scores indicate higher severity of trait anxiety.

Also, participants rated their mood responses after CPR: “to what extent do you feel sad/fearful/shocked about the event?” from 0 (not at all) to 10 (extremely).

#### 3.3.2. Intrusion diary

Intrusive memory was defined as the spontaneous appearance of the CPR scene (not memories that are deliberately recalled), and the content must be image-based (visually) or sound-based (auditory; replay of the scene, including both verbal and nonverbal sound). The diary was divided into three sections (morning, afternoon and evening). Participants were required to mark in the corresponding sections when intrusive memory occurs, and record the content and categories overleaf. The diary was checked to be up-to-date at a fixed time every day. On day 4 and day 7, participants rated their compliance to diaries of the past 72 h (0–10 points: 0 points means completely inaccurate, and 10 points means very accurate).

#### 3.3.3. Reminder of CPR memories

For the memory reminder cue, all participants were asked to think back to the CPR and briefly tell the researcher the worst moments that came to mind (≈1 min).

#### 3.3.4. Vividness and emotionality of CPR memories

Vividness and emotionality of the recalled event were rated respectively on visual analog scales (VASs) from 0 (not at all vivid/unpleasant) to 100 (very vivid/unpleasant). Vividness is defined as “memories that are very clear and detailed”, and emotionality is defined as “memories that give you an unpleasant feeling when you recall them”.

#### 3.3.5. Visuospatial music tapping game

“Ceaseless Music Note (CMN)” (Beijing Muyuan Technology Co., Ltd., Beijing, China) is a visual spatial tapping game played on smartphone app. In the game, when music/songs are being played, there are boxes fall with a stable speed from the top of the screen to the bottom of the screen in four routes and players need to tap on one of the four keys at the bottom of the route when boxes pass the key. The boxes pass the key in a pace conformed to the music, and the more exact the players press the key, the higher they score in the game. The boxes may be blue (needs single press) or green with a trajectory (needs press and horizontal slip to nearby keys). Most of the music in the game are popular songs. The game can also be played with sound off when participants only press the falling boxes without listening. All participants wore headphones whether the sound was on or not. Participants in the music only group simply chose and listened to the listed music in the game but were not allowed to see or tap on the screen.

#### 3.3.6. Devotion to the CMN game

Concentration (How well did you concentrate on the game/music?), enjoyment (How much did you enjoy the game/music?), difficulty (How difficult was the game for you?), and addiction (How much do you want to continue the game/music you just played/listened?) were rated on separate scales from 0 (not at all) to 10 (extremely).

#### 3.3.7. Impact of Event Scale-Revised (IES-R)

The Intrusion subscale of IES-R (52) was applied on Day 7 to assess participants' distress related to the experimental trauma during the past 6 days. The 22-item scale is comprised of 3 subscales representative of the major symptom clusters of posttraumatic stress: intrusion (8 items), avoidance (8 items), and hyperarousal (6 items). Each item is rated from 0 (not at all) to 4 (extremely).

#### 3.3.8. Demand ratings

On day 7, participants were asked to rate how much they believed playing the game/listening to the music 3 days after CPR would increase or decrease the intrusive visual or auditory memories from −10 (extremely decrease), 0 (no impact) to 10 (extremely increase).

### 3.4. Procedure

**Figure 2:**
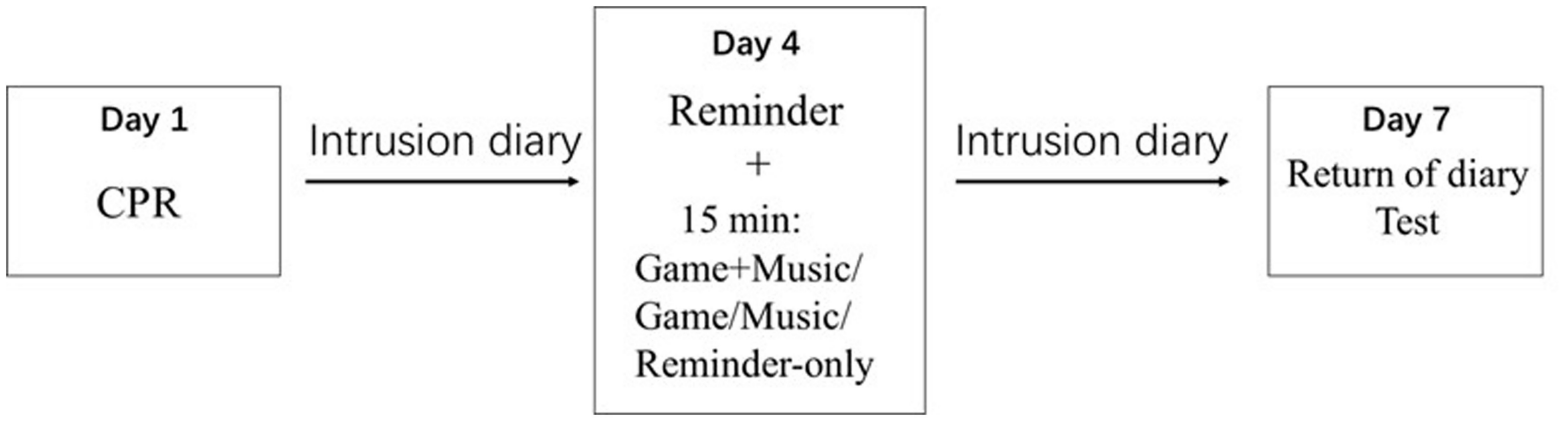
General study design.

Day 1: (Figure 2) Participants were recruited according to inclusion and exclusion criteria, and signed informed consents. Baseline characteristics were recorded: age, BDI-II, STAI-T. Participants rated their mood responses after CPR. Then, participants practiced playing CMN game for 3 min with matching music. They were instructed to note down intrusive memories in the next 72 h.

Day 4: Participants returned to the laboratory, handed in their diaries, and rated their compliance to diaries. Participants were then randomly allocated in a 1:1:1:1 ratio to four parallel treatment conditions [RGM (+), RGM (-), RM (+), and R)]. For the memory reminder cue, all participants were asked to think back to the CPR and briefly told the researcher the worst moment that came to mind (<1 min). Then participants rated the vividness and emotionality of the recalled event respectively. After 5 min' break, the intervention began: participants in the RGM (+) group started game with music; those in the RGM (-) group started game without music; those in the RM (+) group listened to music, but were not allowed to play the tapping game; those in the R group sat quietly for 15 min. Each piece of music (or single game without music) lasted for ~1.5 min. Participants were given several seconds of interval to choose their preferred music to play, and for ~15 min in total. We chose the duration of 15 min because it's an average interventional duration for Tetris game, and many studies have demonstrated its efficacy. Additionally, the CMN game used in our study seemed to be more attracting than Tetris (images more colorful and with pleasant music), so we did not increase the playing time. The difficulty levels were set as “simple” (5 pieces) and “difficult” (4 pieces) and “master” (1 piece). This difficulty ensured that the majority of the boxes could be pressed by the participants but with some difficulty, so that the participants could keep concentrated and feel enjoyable. Participants were encouraged to reach scores as high as they can. After the game/music, participants needed to rate the levels of concentration, enjoyment, difficulty, and addiction. Participants were instructed to note down intrusive memories in the next 72 h.

Day 7: Participants returned to the laboratory, handed in their diaries, and rated their compliance to diaries. They again thought back to the CPR and told it to the researcher, and then rated the vividness and emotionality. Participants were also asked to complete the IES-R scales for the impact of CPR in the past week, and to rate the demand ratings. Finally, all participants were thanked and reimbursed for taking part.

### 3.5. Statistical analysis

Baseline variables (age, BDI-II, STAI-T, sad/fearful/shocked level), diary compliance, game scores, pre-intervention vividness/emotionality of CPR memories, the mean number of pre-intervention intrusions (days 1 to 3), concentration/difficulty/enjoyment/addiction level, demand ratings, IES-R were analyzed using Kruskal-Wallis tests or Mann-Whitney U tests.

Quade test (non-parametric ANCOVA) was used to test between-group comparisons in the mean number of intrusions post-intervention (days 4 to 6) and in the post-intervention vividness/emotionality, followed by planned comparisons (Games-Howell corrected) (49).

Day-to-day differences in the number of intrusions from day 3 to 4 were analyzed using a two-way mixed ANOVA with time as within-subjects factor and group as between-subjects factor.

Statistical analysis was performed by Statistical Package for the Social Sciences (version 19.0 for Windows; SPSS, Chicago, IL, USA). Two-tailed tests were used and a *P* < 0.05 was considered significant.

## 4. Results

### 4.1. Baseline characteristics

Baseline characteristics, diary accuracy, demand ratings, and game score/difficulty of the 76 participants were listed in Table 1. No significant difference of these variables was found among different groups.

**Table 1:** Baseline characteristics (variables were presented as mean ± SD).

**Variables**	**RGM (+)**	**RGM (-)**	**RM(+)**	**R**	**Kruskal-Wallis**	
	**(n**=**20)**	**(n**=**19)**	**(n**=**18)**	**(n**=**19)**	*X* ^2^	***P***
Age	23.65 ± 3.97	25.32 ± 5.03	26.67 ± 6.10	25.21 ± 5.17	2.08	0.56
BDI-II	2.10 ± 1.79	2.16 ± 1.76	2.56 ± 1.54	1.95 ± 1.70	1.97	0.58
STAI-T	34.45 ± 11.98	35.05 ± 8.43	39.11 ± 9.72	38.37 ± 11.04	4.32	0.23
Sad	7.30 ± 1.82	7.42 ± 1.79	7.56 ± 1.67	7.95 ± 1.36	1.33	0.72
Fearful	6.45 ± 1.86	6.53 ± 1.53	5.61 ± 1.77	5.21 ± 2.21	5.00	0.17
Shocked	6.65 ± 1.98	7.58 ± 1.57	6.44 ± 1.64	6.95 ± 1.50	3.87	0.28
Pre-intervention intrusions (total)	12.20 ± 4.62	10.37 ± 3.28	11.89 ± 5.40	14.16 ± 5.62	4.38	0.22
Pre-intervention intrusions (visual)	10.15 ± 3.51	8.55 ± 3.71	8.85 ± 5.21	10.50 ± 4.79	2.34	0.51
Pre-intervention intrusions (auditory)	6.55 ± 4.04	5.00 ± 3.91	6.95 ± 4.78	7.55 ± 5.71	2.09	0.55
Game difficulty	6.60 ± 1.56	7.11 ± 1.07				0.38^*^
Game score	244.00 ± 48.52	259.63 ± 53.73				0.39^*^
Demand ratings	0.70 ± 1.68	−0.16 ± 1.69	−0.11 ± 1.15		2.44	0.30
I diary accuracy	8.00 ± 1.14	7.95 ± 1.05	8.11 ± 0.94	8.42 ± 1.04	2.20	0.53
II diary accuracy	7.95 ± 1.16	8.05 ± 1.15	8.28 ± 1.13	8.47 ± 0.94	2.05	0.56
Pre-intervention vividness	69.70 ± 11.93	73.21 ± 10.44	71.78 ± 9.86	72.90 ± 10.17	1.81	0.61
Pre-intervention emotionality	76.95 ± 8.65	78.89 ± 10.71	80.39 ± 8.54	75.95 ± 9.47	2.69	0.44

* Mann-Whitney U tests.

### 4.2. Primary outcomes

**Figure 3:**
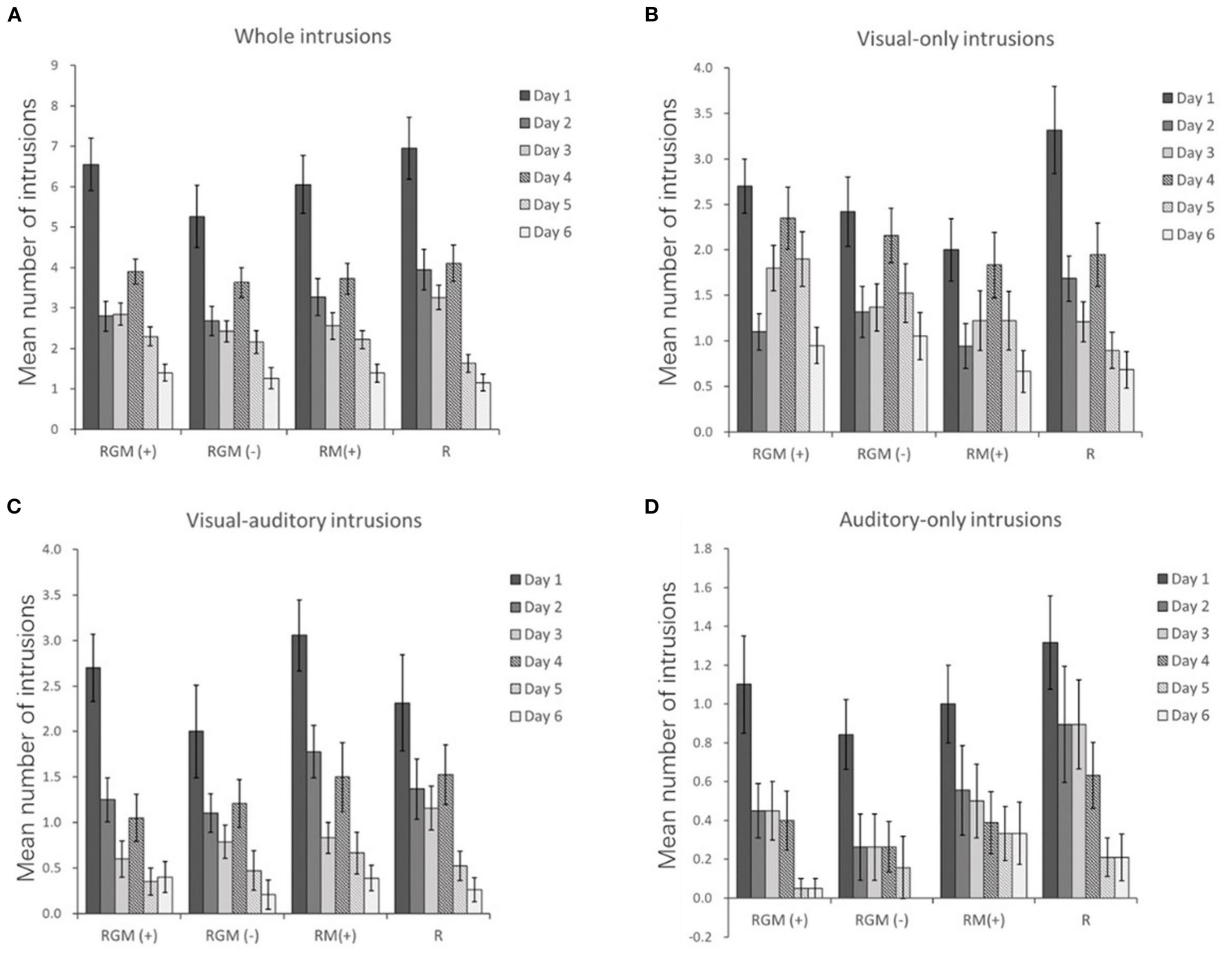
Trajectory of mean number of whole **(A)**, visual-only **(B)**, visual-auditory **(C)**, and auditory-only **(D)** intrusions. Error bars depict SEMs.

The mean number of intrusions on each day were shown in Figure 3. The Quade test and one-way ANOVA test of unstandardized residuals of a regression analysis (number of intrusions before intervention on number of intrusions after intervention) showed no significant effect of intervention type on numbers of total intrusions or visual or auditory intrusions (M_RGM(+)_ = −4.60 ± 5.32, M_RGM(−)_ = −3.32 ± 4.58, M_RM(+)_ = −4.56 ± 4.79, M_R_ = −7.26 ± 6.05, F_3,72_ = 0.35, *P* = 0.79, η^2^ = 0.014 for total intrusions; F_3,72_ = 0.63, *P* = 0.60, η^2^ = 0.026 for visual intrusions; F_3,72_ = 0.82, *P* = 0.49, η^2^ = 0.033 for auditory intrusions).

To analyze the number of intrusions from day 3 to day 4 between groups, a two-way mixed ANOVA revealed a significant main effect of time (F_1,43.23_=19.77; *P*<0.001; η^2^ = 0.12), but no significant main effect of group (F_3,3.20_ = 1.46; *P* = 0.23; η^2^ = 0.03) or a time×group interaction (F_3,0.26_ = 0.12; *P* = 0.95; η^2^ = 0.002).

**Figure 4:**
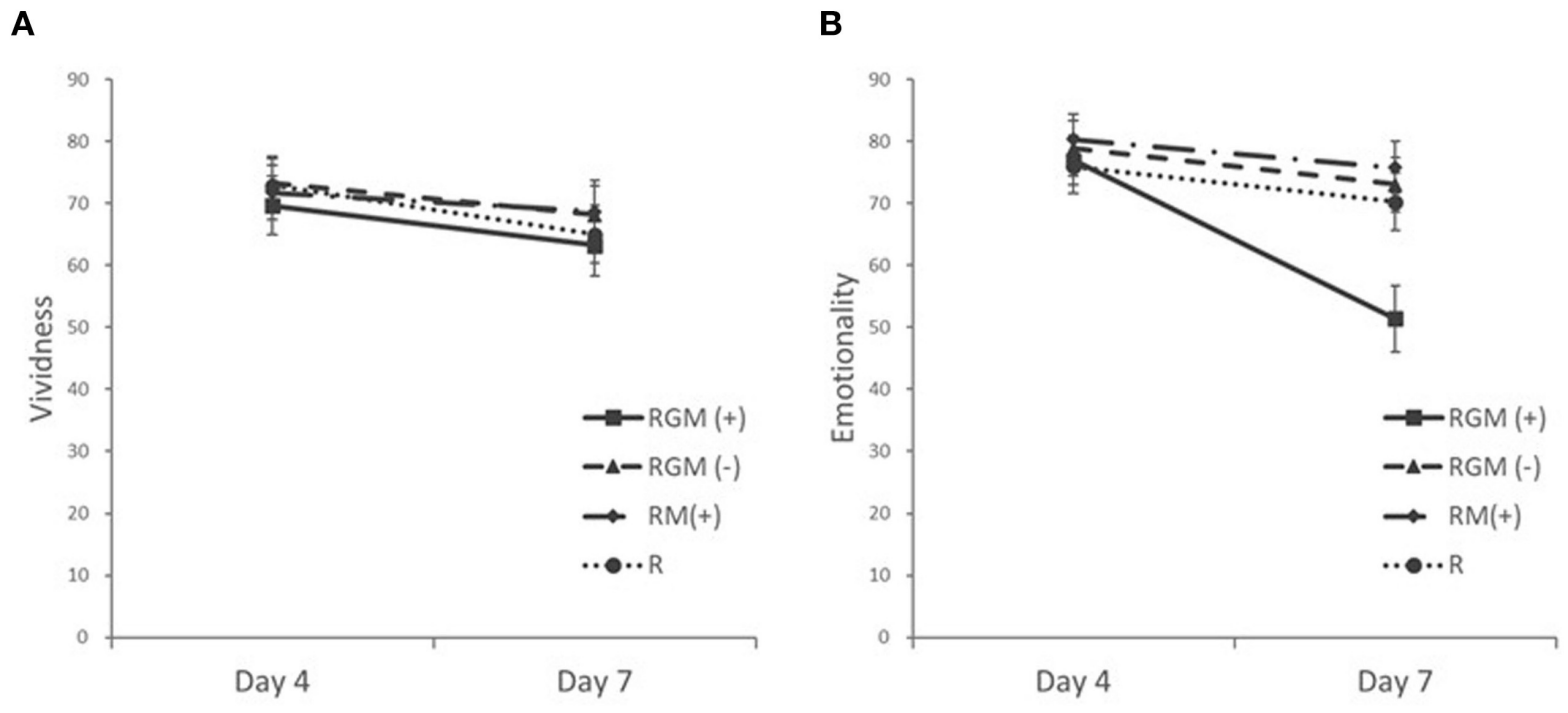
Mean vividness **(A)** and emotionality **(B)** ratings at pre-intervention (Day 4) and post-intervention (Day 7). Error bars depict SEMs.

The Quade test and one-way ANOVA test of unstandardized residuals of a regression analysis (vividness before intervention on vividness after intervention) showed no significant effect of intervention type on the degree of vividness (M_RGM(+)_ = −6.45 ± 7.72, M_RGM(−)_ = −5.00 ± 8.78, M_RM(+)_ = −3.06 ± 7.56, M_R_ = −7.84 ± 6.85; F_3,72_ = 1.97, *P* = 0.13, η^2^ = 0.076; Figure 4A).

The Quade test and one-way ANOVA test of unstandardized residuals of a regression analysis (emotionality before intervention on emotionality after intervention) showed a significant effect of intervention type (M_RGM(+)_ = −25.55 ± 13.59, M_RGM(−)_ = −5.84 ± 7.78, M_RM(+)_ = −4.56 ± 8.70, M_R_ = −5.63 ± 7.33; F_3,72_ = 17.76, *P*<0.001, η^2^ = 0.425; Figure 4B). Planned comparisons (Games-Howell) between all groups showed that the RGM (+) group had a significantly larger decrease in emotionality after intervention compared to the RGM (-) group (Mean difference_Residuals_ = −26.47, SE = 4.73, *P*<0.001), the RM (+) group (Mean difference_Residuals_ = −29.63, SE = 5.08, *P*<0.001) and the R group (Mean difference_Residuals_ = −25.32, SE = 4.93, *P* < 0.001).

No significant difference of IES-R intrusions subscale scores was found between the four groups (M_RGM(+)_ = 9.65 ± 4.63, M_RGM(−)_ = 8.79 ± 4.86, M_RM(+)_ = 7.89 ± 3.20, M_R_ = 9.11 ± 5.11; *X*^2^ = 1.03, *P* = 0.79).

### 4.3. Concentration/enjoyment/addiction ratings

Kruskal-Wallis tests showed significant difference of concentration (*X*^2^ = 10.58, *P* = 0.01), enjoyment (*X*^2^ = 29.15, *P* < 0.001), and addiction (*X*^2^ = 22.95, *P* < 0.001) ratings among the three groups. Mann-Whitney U tests further showed significant difference of enjoyment (M_RGM(+)_ = 8.45 ± 1.24, M_RGM(−)_ = 5.42 ± 1.35, *P*<0.001) and addiction (M_RGM(+)_ = 7.15 ± 1.80, M_RGM(−)_ = 3.47 ± 1.98, P<0.001) ratings between RGM(+) group and RGM(-) group, and significant difference of concentration (M_RGM(+)_ = 8.40 ± 1.20, M_RM(+)_ = 7.00 ± 1.25, *P* = 0.002), enjoyment (M_RM(+)_ = 7.06 ± 1.09, *P* = 0.001), and addiction (M_RM(+)_ = 5.39 ± 1.64, *P* = 0.004) between RGM(+) group and RM(+) group. Mann-Whitney U tests also showed significant difference of concentration (M_RGM(−)_ = 7.84 ± 1.14, *P* = 0.049), enjoyment (M_RGM(−)_ = 5.42 ± 1.35, *P* = 0.001), and addiction (M_RGM(−)_ = 3.47 ± 1.98, *P* = 0.006) between RGM (-) group and RM (+) group.

## 5. Discussions

Previous studies rarely measured the vividness/emotionality ratings and intrusive memories at the same time (19, 28), and visuospatial game play was generally with sound-off (53). As far as we know, the present study was the first to show that the game-matching background music can have an add-on effect for single tapping game with no sound in reducing the emotionality of previous aversive memories. However, we did not find a positive effect in reducing the number of intrusions or the vividness of aversive memories by playing the visuospatial music tapping game CMN applied at 3 days after CPR.

### 5.1. Reducing vividness and emotionality

Implementing dual tasks can reduce the vividness and emotionality of aversive memories, such as eye movement desensitization and reprocessing (EMDR) therapy (54–56), drawing complex figures (57), and mental arithmetic (27). The underlying mechanism is assumed to be the working memory (WM) theory. According to this theory, the aversive memory can be affected upon recalling when one is doing another task, which taxes one's limited working memory capacity (58). Playing Tetris game has also been suggested to involve both storage and processing resources within visuospatial WM (14), and there were also studies showing that playing Tetris could reduce the vividness and emotionality of aversive memories (59) or cravings (60).

Music therapy has been suggested as a viable and effective treatment for improving PTSD symptoms (61), and it was effective in reducing the physiological signs of agitation and anxiety (62–69). It was suggested that music can help individuals improve their ability to both express and regulate emotions (70), and help evoke vivid autobiographical memories (71). In the military population, active music making such as drumming was considered most beneficial (70). Although the music therapy lacks a standard protocol, there were studies indicating a general rule to consider is preferred music lasting 20–30 uninterrupted minutes, at least twice daily, in a comfortable position and environment (72, 73). When the music is simple, familiar to the patient, and contains 60–80 beats per minute, it is most effective (62).

In our study, we did not use dual tasks paradigm but intervened after a short retrieval of aversive memories. The game scores were not significantly different between group RGM (+) and group RGM (-), which suggests that the background music exerted no additional cognitive taxing on players. Thus, it is unlikely that the effect of RGM (+) group in reducing emotionality attributed to the working memory taxed by background music. The background music did not seem to meet the standard of music therapy, since it is relatively fragmented (about 10 pieces), short (totaling 15 min) and applied only once. In our study, no effect in reducing emotionality was found for background music only [group RM (+)] or game play only [group RGM (-)]. So, it is also unlikely that the effect of gameplay plus background music in the group RGM (+) is due to the simple summation effect of two methods. As expected, the RGM (+) group had the highest ratings in concentration, enjoyment and addiction among the groups. These accorded with the feature of the so-called flow experience (74), which seemed to be the underlying mechanism for the add-on effect of background music. Our results showed a reduction of emotionality but not vividness in the group RGM (+), which is different from methods such as EMDR which can lower both emotionality and vividness. We assumed two possibilities for this discrepant effect of the gameplay plus background music. One is due to the different mechanism of reconsolidation and dual tasks, and the other is that the emotional aspect of aversive memories is easier to be influenced than declarative aspect (75).

### 5.2. Reducing the numbers of intrusive memories

As for visuospatial games, although it has been repeatedly reported that playing Tetris game can reduce the number of intrusions in experimental conditions (19, 31, 49, 53, 76, 77), real-world traumas (20, 21, 75, 78), and PTSD patients (22), and in either the consolidation (1920, 31, 73) or the reconsolidation time window (22, 49, 53, 77), there were some studies finding null results with Tetris game (30) or visuospatial tasks either quite similar to (28) or different from (28, 29) Tetris. One of the important reasons they attributed to for the null results was the possibility of less interesting and attention-grabbing of games, which is the basis of flow state.

Previous studies mostly assessed only visual intrusions or only auditory intrusions, and the intervention was also visually or auditorily focused. In our study, we assessed both visual and auditory intrusions. In the hospital settings, nurses experienced CPR have both visual and auditory intrusive memories, and some of those were combined. However, we did not find a positive effect of reducing the number of intrusive memories in any modality by CMN game. It is interesting to reveal the relationship between emotionality and intrusion number. The two were expected to be positively correlated. One explanation for our contradictory results is that the number of intrusions decreased rapidly in all groups, and the ceiling effect prevents us from finding a positive results. The second explanation is that the emotionality may be more susceptible than intrusions by the intervention of playing visuospatial games. As listed above that some previous studies also showed negative results for reducing intrusions, on the contrary, studies showing negative results for reducing emotionality are rare. In the study by Holmes et al. (19), results showed that playing Tetris game did not reduce emotionality immediately after intervention compared with no tasks. Similarly, in another study by Asselbergs et al. (28), results also revealed no significant difference between playing visuospatial games and no tasks at 7 days post-intervention. In both studies, the scholars used trauma film as an experimental analog, and the mood ameliorated rapidly in all groups. So, there may be a ceiling effect: in the former study, Tetris-playing group did show better (although insignificant) mood condition after intervention than no-task group in two separate experiments (mood assessment in experiment 1: 42.3 vs. 58.1% of full scores; experiment 2: 37.8 vs. 47.6% of full scores); in the latter study, dual tasks groups (playing game whilst recalling aversive film memory) also showed better (although insignificant) emotionality scores than no task control group in two separate experiments.

### 5.3. Clinical implications

It has been reported that careers can be facilitated by improving resistance to chronic stress (79). In the clinical settings, anxiety is common among both patients (80, 81) and doctors (82), and relaxing music is beneficial to surgery (82) and cardiovascular disease (81). ICU nurses are frequently exposed to traumatic events while providing care to vulnerable patient populations. Playing visuospatial game CMN may be a simple and accessible way to ameliorate the distress emotions. It may be helpful for improving nursing career satisfaction and reducing turnover rate. Furthermore, wider applications of this self-help therapy can be expected for individuals exposed other traumatic events, especially those causing massive casualties (e.g., natural disasters and wars), when psychologists are lacking.

Since our results indicate flow experience as a key boundary condition for successful intervention, future study may focus on whether the effect is game modality-specific or any kind of game can have this effect as long as the player is immersed in the playing. Additionally, it is also interesting to investigate whether playing Tetris can reduce the number of intrusive memories for these populations.

### 5.4. Limitations

First, since reconsolidation intervention may have different effects on female and male participants (83), we only recruited female participants, which constitute the majority of nurse population. Thus, the effect of playing CMN games can not necessarily be translated to males; second, considering that this video game may not attract elder players, and elder nurses may have different mental status compared to younger ones (e.g., due to different work experience and endocrine changes), the participants in our study were relatively young, all under the age of 41. So, whether this intervention method is suitable for elder players and has similar effects needs further study. Third, since flow experience could not be evaluated by objective methods, and it's only a potential explanation of the results. We cannot rule out the possibility that other mechanisms underlie the effect. These problems are to be solved in the future.

## 6. Conclusions

As far as we know, the present study was the first to show that the game-matching background music can have an add-on effect for single tapping game with no sound in reducing the emotionality of previous aversive memories. We proposed that flow experience as a key boundary condition for successful reconsolidation intervention. With the advent of virtual reality (VR) technique featuring immersive experience, the combination of VR with behavioral therapies has broad prospects in the realm of psychology and deserves extensive study.

## Data availability statement

The original contributions presented in the study are included in the article/supplementary material, further inquiries can be directed to the corresponding authors.

## Ethics statement

The studies involving human participants were reviewed and approved by the Ethics Committee of General Hospital of Southern Theatre Command. The patients/participants provided their written informed consent to participate in this study.

## Author contributions

XZ, ZQ, and CJ designed the research. CJ and WC analyzed the data and wrote the manuscript. JW, KC, and YZ collected the data. LT draw the figures and critically reviewed the manuscript. XZ approved the final manuscript. All authors contributed to the article.
